# Severe mitral valve papillary muscle rupture of isolated Whipple's endocarditis: a case report and review of the literature

**DOI:** 10.3389/fcvm.2025.1669997

**Published:** 2025-11-11

**Authors:** Yan Shi, Xinchen Wang, Xinxin Mao, Liangyu Mi, Wanglin Liu, Na Wang

**Affiliations:** 1 Department of Critical Care Medicine, Peking Union Medical College Hospital, Peking Union Medical College, Chinese Academy of Medical Science, Beijing, China; 2 Department of Pathology, Peking Union Medical College Hospital, Peking Union Medical College, Chinese Academy of Medical Science, Beijing, China

**Keywords:** tropheryma whipplei, Whipple's disease, endocarditis, culture-negative, metagenomic next-generation sequencing, case report

## Abstract

**Background:**

*Tropheryma whipplei* endocarditis (TWE) is rarely reported. Diagnosis is particularly challenging when it occurs as isolated TWE without classical manifestations of Whipple's disease.

**Case presentation:**

A 35-year-old Asian female with systemic lupus erythematosus presented with acute heart failure secondary to mitral valve papillary muscle rupture as her sole symptom, requiring emergent veno-arterial extracorporeal membrane oxygenation support and urgent valve replacement. Intraoperative absence of vegetations and negative conventional microbiological examination preliminarily ruled out infective endocarditis. However, on postoperative day (POD) 3, her condition rapidly deteriorated into septic shock. Follow-up chest CT revealed bilateral asymmetric pulmonary infiltrates inconsistent with cardiogenic pulmonary edema alone. Metagenomic next-generation sequencing (mNGS) of bronchoalveolar lavage fluid detected *T. whipplei*, providing a crucial diagnostic breakthrough. Subsequent periodic acid-Schiff staining of the resected valve confirmed the definitive diagnosis of isolated TWE. Targeted meropenem therapy for 5 days resulted in significant improvement in both pneumonia and septic shock, permitting ECMO discontinuation. The patient was successfully extubated by POD 12 and discharged on POD 22 with oral co-trimoxazole and doxycycline in a stable condition.

**Conclusion:**

We present the first case of isolated TWE in a young Asian female, notable for its atypical clinical presentation, fulminant progression, and profound diagnostic challenges. Clinicians should maintain a high vigilance for blood culture–negative endocarditis. Timely diagnosis and appropriate treatment are crucial for improving prognosis. mNGS analysis of samples from suspected disseminated sites may yield crucial diagnostic breakthrough.

## Introduction

Whipple's disease (WD) is a rare systemic disease caused by the gram-positive bacillus *Tropheryma whipplei*. This bacterium is a conditionally pathogenic commensal organism often detected in environments such as sewage and soil. Its exact pathogenesis remains undetermined. First described by George Hoyt Whipple in 1907, its annual incidence is approximately 1 per million, predominantly affecting Caucasian males, with a mean age of onset around 50 years (1, 2). WD is classically characterized as a chronic and consuming disease, with hallmark manifestations including gastrointestinal symptoms (abdominal discomfort, diarrhea, malabsorption), weight loss and arthralgias, collectively referred to as classical WD (2). In 1997, *T. whipplei* was first identified as a causative agent of blood culture-negative endocarditis (BCNE) (3). Since then, *T. whipplei* endocarditis (TWE) has been reported in the America and Europe, accounting for 2.6% to 6.3% of BCNE cases (4–6). The vast majority of cases manifest classic features of WD, while isolated TWE — defined as valvular infection without systemic symptoms of classical WD— remains extremely rare (3, 7, 8). Its diagnosis is particularly challenging as it frequently fails to meet Duke's diagnosis criteria for infective endocarditis (IE). Furthermore, this fastidious pathogen exhibits inherent resistance to conventional microbial culture techniques, necessitating specialized diagnostic methods (e.g., molecular testing, histopathological special staining) (1–3). Consequently, diagnosis is often delayed, increasing the risk of severe complications such as embolism, heart failure, valve damage, or even death (9, 10). We herein report the first case of isolated TWE in a young Asian female. This case highlights its atypical manifestations, rapid deterioration, and profound diagnostic challenges. We hope this report provides valuable insights for the diagnosis and management of similar cases in the future.

## Case presentation

A 35-year-old female presented to the emergency department with a 3-day history of palpitations and chest tightness. Her history was significant for systemic lupus erythematosus with lupus nephritis, initially diagnosed 30 years ago due to fever and malar rash, with development of microscopic hematuria and mild proteinuria over a decade ago. Her last follow-up six months ago showed clinical stability with normal serum creatinine, autoimmune antibody and complement profile, and no evidence of other systemic involvement (e.g., joints, hematopoietic system, or heart), while being maintained on prednisone monotherapy at 5 mg daily.

**Figure 1:**
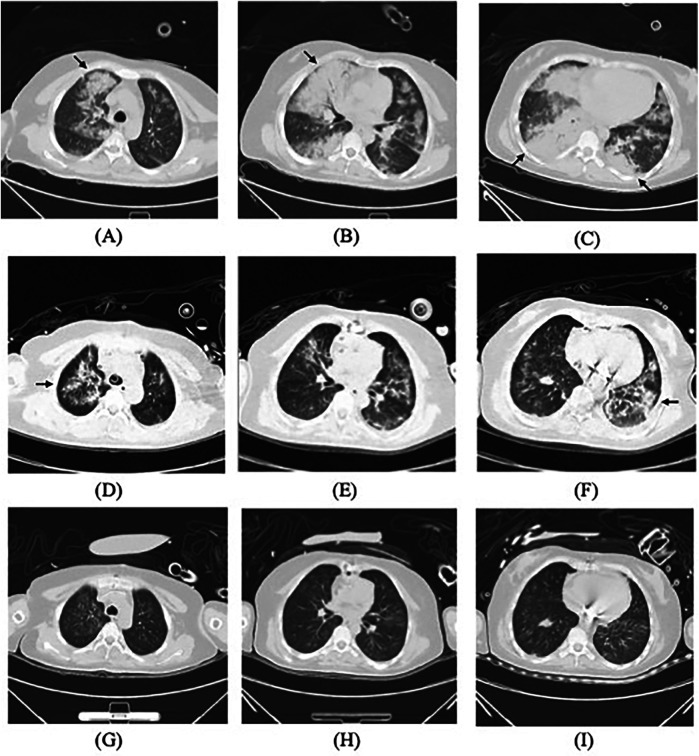
Lung CT scans: **(A–C)** on admission, **(D–F)** on the postoperative day 3, and **(G–I)** almost one week after initiating targeted treatment, demonstrating gradual resolution of pulmonary infiltrates (arrows).

**Figure 2:**
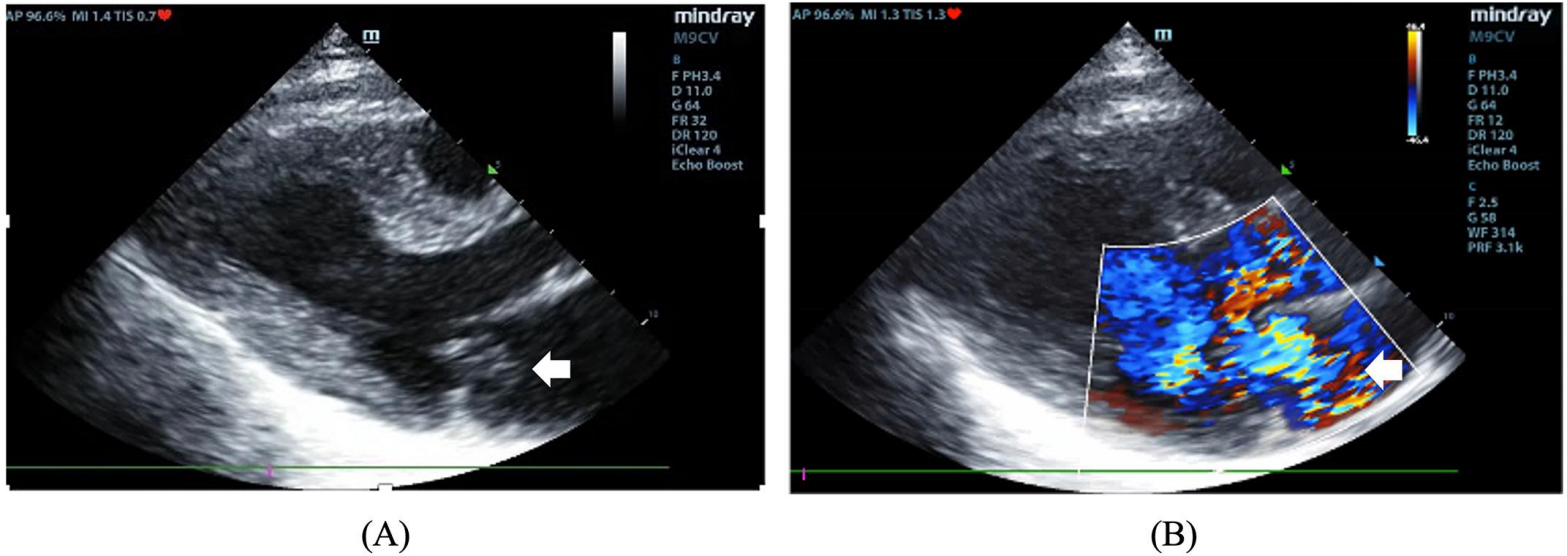
**(A)** The two-dimensional image showing a mass protruding into the left atrium (arrow). **(B)** Color Doppler image showing severe mitral regurgitation.

On admission, she exhibited signs of acute left heart failure. Physical examination documented a temperature of 36.3°C, tachycardia (145 bpm), hypotension (92/52 mmHg), tachypnea (28 breaths/min) with oxygen saturation 88% on room air. Widespread bilateral pulmonary crackles and an apical gallop rhythm with a grade III/VI holosystolic murmur at the mitral area were noted. Blood tests revealed elevated levels of C-reactive protein (37.5 mg/L, normal < 3 mg/L) and N-terminal B-type natriuretic peptide (12,855 pg/mL, normal < 125 pg/mL), while white blood cells (WBC) (8.12 × 10^9^/L), platelet (257 × 10^9^/L), hemoglobin level (143 g/L), erythrocyte sedimentation rate (16 mm/h), and hepatic and renal function were all normal. Chest computed tomography (CT) showed extensive bilateral pulmonary infiltrates with significant consolidation (Figures 1A–C). The electrocardiogram indicated supraventricular tachycardia at 164 bpm with 1 mm ST segment depression in leads V1 to V6. Transthoracic echocardiography revealed mitral valve papillary muscle rupture and a suspicious mass-like lesion protruding into the left atrium (Figures 2A,B). Emergent coronary computed tomography angiography demonstrated chronic occlusion of the left anterior descending artery, with retrograde perfusion supplied by collateral vessels originating from the circumflex artery, effectively excluding acute coronary syndrome.

The patient developed progressive hypoxemic respiratory failure with pink, frothy sputum production, necessitating endotracheal intubation and transfer to the intensive care unit (ICU). Upon ICU admission, she demonstrated hyperpyrexia (41°C), severe pulmonary edema requiring 100% FiO_2_, and refractory shock requiring massive, supra-conventional doses of norepinephrine (NE) tartrate infusion (at 2 µg/kg/min; pure base: tartrate ≈ 1:2). Given suspicion of IE, two sets of blood cultures were collected, and empirical broad-spectrum antimicrobial therapy with meropenem plus vancomycin was initiated. Simultaneously, veno-arterial extracorporeal membrane oxygenation (ECMO) was emergently instituted, followed by urgent mitral valve replacement surgery. Intraoperative findings confirmed papillary muscle rupture with complete destruction of the posterior mitral leaflet, without evidence of vegetations or abscess formation.

**Figure 3:**
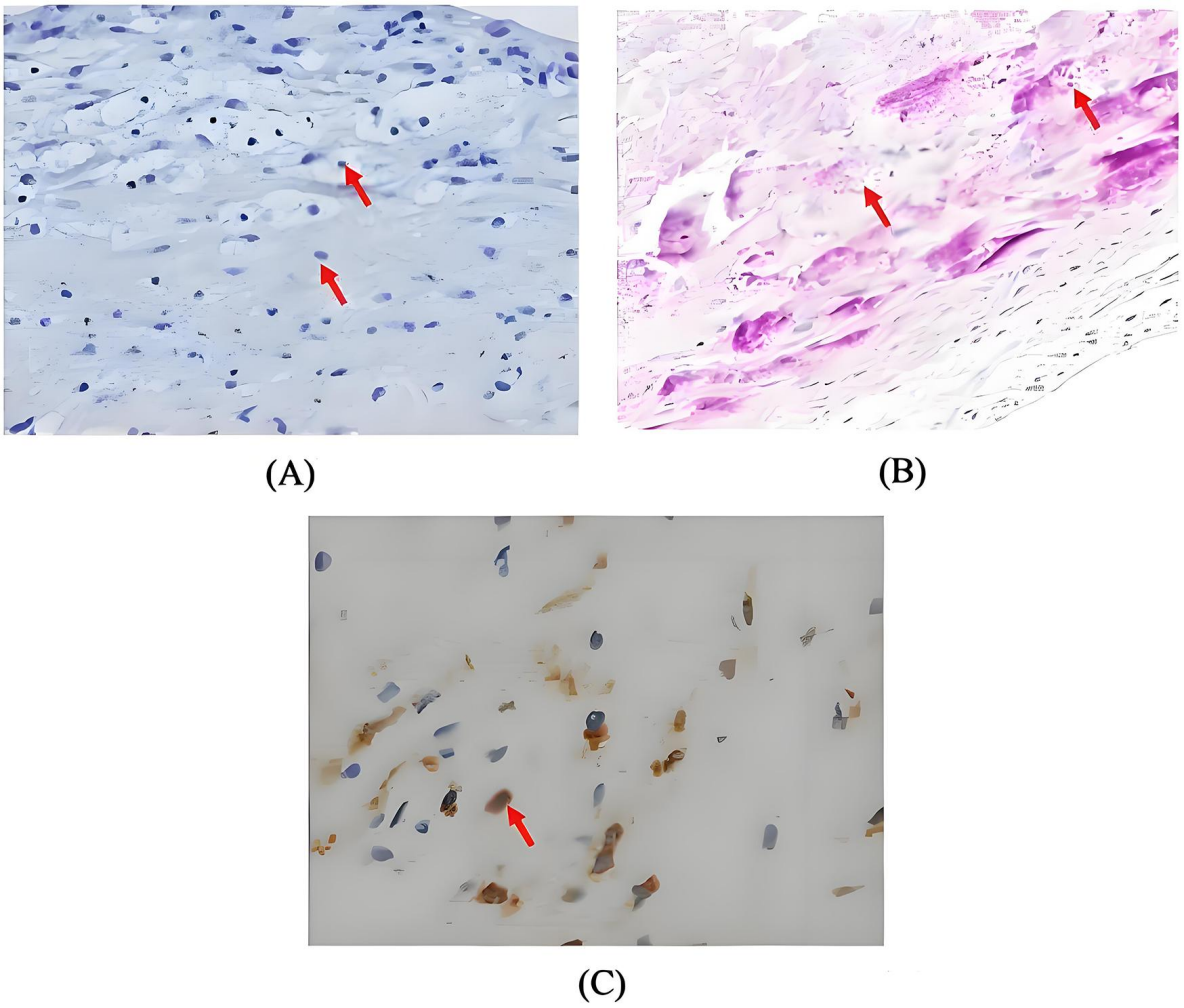
**(A)** Haematoxylin and eosin stain demonstrating foamy macrophage infiltration (magnification × 200, arrow); **(B)** periodic acid-schiff stain revealing characteristic intracellular bacilli (magnification × 200, arrow); **(C)** CD68 immunohistochemistry highlighting histiocytic aggregation (magnification × 400, arrow).

**Figure 4:**
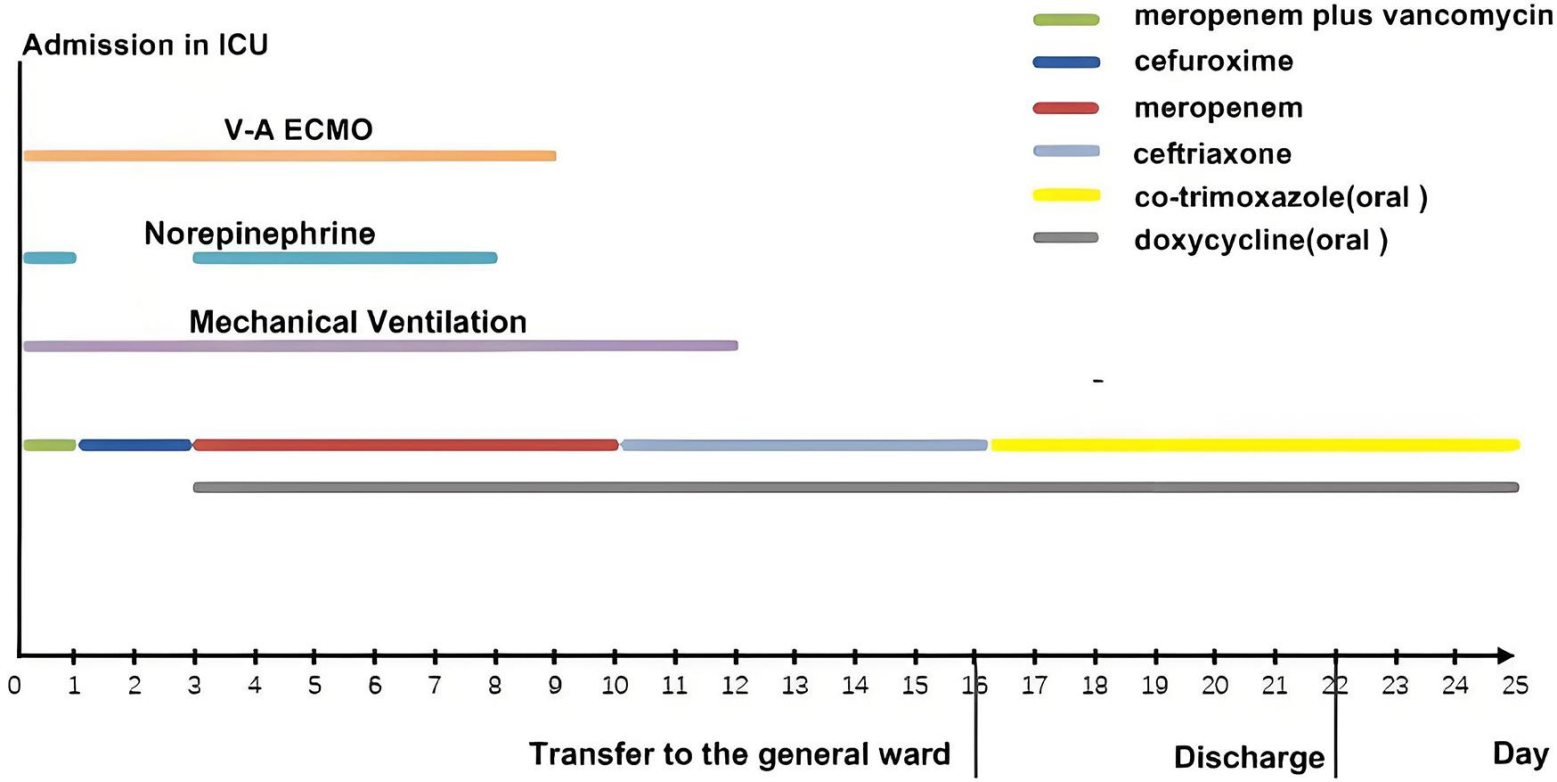
The timeline and treatment course for this patient.

Postoperatively, the patient maintained hemodynamic stability on ECMO support without vasoactive drugs. Intraoperative absence of valvular vegetations combined with negative extensive microbiological investigations (including blood cultures, serological assays, and valve tissue smear/cultures) preliminarily excluded IE. Consequently, antimicrobial therapy was de-escalated to cefuroxime. However, on postoperative day (POD) 3, the patient developed septic shock, necessitating reinitiation of NE infusion (0.3–0.4 µg/kg/min). Her clinical manifestations included fever (39°C), leukocytosis (WBC increased from 8.75 to 19.47 × 10^9^/L), and markedly elevated inflammatory biomarkers: procalcitonin rising from 0.46 to 21 ng/mL (normal < 0.05 ng/mL) and IL-6 > 1,000 pg/mL (normal < 5.9 pg/mL). Follow-up chest CT revealed asymmetrical peribronchial infiltrates with progression in the right upper lobe compared to preoperative imaging (Figures 1D–F), These radiographic patterns were inconsistent with cardiogenic pulmonary edema. Given the atypical disease progression and strong suspicion for occult infection, a comprehensive microbiological reevaluation was undertaken, including repeat blood and tracheal aspirate (TA) cultures, and metagenomics next generation sequencing (mNGS) of paired blood and bronchoalveolar lavage fluid (BALF) specimens. Notably, BALF mNGS detected *T. whipplei* as the dominant pathogen (266,358 sequence reads and 99.7% relative abundance), whereas blood mNGS, blood culture and TA culture remained consistently negative. These findings strongly suggested WD with pulmonary involvement. Subsequent histopathological reassessment of valve tissue definitively confirmed TWE based on characteristic features displayed by special staining techniques, including Hematoxylin and Eosin showing foamy macrophage infiltration, CD68 immunohistochemistry confirming histiocytic lineage, and periodic acid-Schiff (PAS) highlighting intracellular bacilli (Figures 3A–C). Antimicrobial therapy was escalated to meropenem (1 g IV q8 h) plus oral doxycycline (100 mg twice daily). Following 5 days of targeted antimicrobial therapy, chest CT showed significant resolution of pulmonary infiltrates (Figures 1G–I), with resolution of septic shock permitting ECMO discontinuation on POD 9. One week later, meropenem was switched to ceftriaxone (2 g IV qd) following normalization of temperature and WBC. The patient was successfully extubated on POD 12 and discharged from the ICU on POD 16. Upon targeted history-taking, she specifically denied classical WD symptoms, such as diarrhea, abdominal pain, weight loss, or arthralgias. She was discharge on POD 22 with continuation therapy comprising doxycycline and co-trimoxazole (Figure 4). At 3-month follow-up, the patient was in good condition. Given her persistent immunocompromised status, indefinite co-trimoxazole suppression therapy was recommended.

## Discussion

We reported the first case of isolated TWE in a young Asian female. The patient presented with acute, life-threatening mitral valvular dysfunction secondary to papillary muscle rupture, requiring emergent ECMO support and valve replacement. Notably, Intraoperative absence of vegetations and negative comprehensive microbiological workup effectively excluded IE at initial assessment. However, rapid postoperative clinical deterioration prompted thorough investigation. Ultimately, mNGS of BALF identified *T. whipplei,* providing a crucial diagnostic breakthrough. Subsequent targeted histopathological staining of valve tissue definitively confirmed isolated TWE. This case underscores unique clinical manifestations of this rare disease and proposes potential diagnostic approaches for early recognition.

Systematic research on isolated TWE remains limited. Building upon a 2010 review (11) —which incorporated literature published through June 2009 and identified 10 isolated TWE cases among 97 total cases — we conducted an expanded Pubmed search on case reports published between July 2009 and December 2024, using search terms “Whipple's” OR “tropheryma” OR “*Tropheryma whipplei"* AND “endocarditis”. Articles were screened by title and abstract for relevance. Full texts of potentially eligible publications were retrieved and re-evaluated against predefined inclusion criteria (the literature search method is detailed in Supplementary File). Isolated TWE was defined by the absence of the clinical manifestations of classic WD (e.g., gastrointestinal symptoms, arthralgia, or unintentional weight loss) and, if available, a negative intestinal biopsy for *T. whipplei.* The screening process was performed by the authors (Y.S. and X.C.W.). Thirty-seven isolated TWE cases from 21 publications were ultimately included (5, 6, 11–29). The flowchart of case selection is shown in Supplementary Figure S1, and the clinical characteristics are summarized in Supplementary Table S1.

All 37 reported cases originated from Europe or North America, with a mean age of 62 years. Males accounted for 89% of cases (*n* = 33). None fulfilled Duke's criteria for IE. The most common acute symptoms were heart failure (57%, *n* = 21), followed by acute ischemic stroke (22%, *n* = 8), peripheral arterial embolism (11%, *n* = 4), acute coronary syndrome (8%, *n* = 3) and non-cardiogenic shock (3%, *n* = 1). Vegetations were present in 75% of cases (*n* = 28). Native valve involvement occurred in 92% (*n* = 34) of cases. Isolated valve involvement frequencies decreased as follows: aortic valve (62%, *n* = 23), mitral valve (16%, *n* = 6), and tricuspid valve (3%, *n* = 1). Aortic and mitral multivalvular involvement was documented in 7 cases. Among 37 patients, TWE was confirmed in 35 cases through 16S rDNA PCR or PAS staining of resected valves, while the remaining 2 patients (nos.11 and 18) who did not undergo valve replacement were diagnosed via PCR detection of *T. whipplei* in pleural effusion (22) and thrombus aspirates (27), respectively. Notably, all 12 cases undergoing duodenal biopsies tested negative for *T. whipplei*. Diverse antibiotic regimens included ceftriaxone, co-trimoxazole, doxycycline, hydrochloroquinine, gentamicin, amoxicillin and vancomycin. Four patients (10.8%; nos.4,15,19 and 23) died shortly postoperatively, with diagnosis confirmed postmortem (12, 23, 26, 29).

The diagnosis of isolated TWE poses significant challenges. As demonstrated in our literature review, systemic manifestations of infection are typically mild or absent, with less than 20% of cases exhibiting fever. Notably, vegetations are not prerequisite for diagnosis, as one quarter of cases lack this finding. Our case exemplifies the diagnostic challenge posed by the absence of systemic manifestations and vegetations, nearly leading to missed diagnosis. While classic WD diagnosis relies on special testing (e.g., PAS stain or PCR) of duodenal biopsy specimens, this approach is ineffective for isolated TWE without gastrointestinal involvement (30). As evidenced in our literature review, all cases undergoing duodenal biopsies yielded negative results. The diagnosis of TWE primarily relies on specialized testing of resected valvular tissue, leading to frequent diagnostic delays until post-valve surgery. Furthermore, when specialized detection techniques are unavailable, it may also lead to missed diagnosis. Recent evidence supports diagnosis via PCR of extracardiac specimens such as pleural fluid (22), thrombus aspirate (27), whereas blood PCR demonstrates consistently low sensitivity (31). Our case corroborated these findings: the initial diagnostic clues for TWE emerged from BALF-mNGS, highlighting the importance of investigating extravalvular dissemination sites. The advent of BALF-mNGS provides clinicians with an effective tool for detecting *T. whipplei* (32–34). Nevertheless, the pathogenic role played by *T. whipplei* in pneumonia remains a nebulous enigma (35). Although it may contribute to aspiration pneumonia pathogenesis alongside other oral flora constituents (35, 36), studies also revealed surprisingly high colonization rates of *T. whipplei* in the lung (37, 38). Therefore, detecting *T. whipplei* via BALF-mNGS may provide important diagnostic clues for WD, but distinguishin its true role in pulmonary presentations (colonization vs. infection) requires careful interpretation. In this case, compelling microbiological evidence, coupled with imaging findings and a favorable therapeutic response, strongly suggests the diagnosis of concurrent severe *T. whipplei-*associated pneumonia. However, the lack of other dominant oral flora in the BALF mNGS results complicates the precise pathogenic elucidation, warranting further investigation. Notably, existing evidence demonstrates that when *T. whipplei* is detected as the sole pathogen in BALF from pneumonia patients, it actually reinforced its true pathogenicity (35).

Sepsis and septic shock represent extremely rare but catastrophic complications of TWE. Current evidence indicates that while a very small proportion (4.5%) of TWE patients develop concomitant sepsis, this complication serves as an independent predictor of poor prognosis (9). Our literature review further confirms that all four patients who progressed to sepsis ultimately died, with autopsy findings from two cases (nos.15 and 23) showing multiple septic emboli affecting multiple organs (23, 29). The mechanisms driving perioperative acute deterioration remain poorly elucidated, our study provides the first documented evidence of IL-6 surge during acute symptom exacerbation. IL-6 is well recognized as a “double-edged sword” in inflammation regulation; blockade of IL-6 signaling may impair pathogen clearance, potentially explaining the severe *T. whipplei* infections observed in patients receiving IL-6 receptor blocker tocilizumab (39, 40). Conversely, excessive proinflammatory cytokine production can induce tissue injury, as exemplified by COVID-19-associated hyperinflammation and multiorgan failure. Thus, sepsis is understood as a dysregulated host response to infection that thereby leads to life-threatening organ dysfunction (41). The relationship between immunosuppression and susceptibility to *T. whipplei* infection remains incompletely understood (42); however, immunosuppression may accelerate clinical deterioration (7, 39, 40). In our literature review, only 8% (*n* = 3, nos. 3, 19 and 23) had documented immunosuppression. Yet, the majority of patients progressing to sepsis (3 of 4 cases) either had underlying immunosuppression (nos. 19 and 23) or chronic alcohol abuse history (no.4). These findings indicated that immunosuppression may contribute to rapid clinical deterioration. Given the high mortality of *T. whipplei* - associated sepsis, heightened clinical vigilance is warranted, particularly in immunocompromised patients. The immune pathway driving the inflammatory storm in our patient may represent a critical direction for future investigation.

This study has several limitations. First, intestinal biopsy for exclusion of classical WD was relatively contraindicated given the patient's critical status. Moreover, obtaining intestinal specimens proves significant challenging in asymptomatic cases**.** Second, specialized diagnostic modalities for *T. whipplei* detection were not routinely available at our institution, potentially delaying pathogen identification. Importantly, pathogen specific diagnostic approaches for BCNE remain predominantly unavailable in resource-limited settings.

## Conclusion

Isolated TWE represents a rare yet life-threatening disease that may even experience acute and rapid perioperative progression. Failure to recognize timely leads to poor prognoses. Therefore, maintaining clinical vigilance for this entity is essential. In cases with high clinical suspicion of BCNE, early initiation of pathogen-specific diagnostic testing is critical for prompt diagnosis and effective management. When invasive valve sampling is unfeasible, specialized analysis of specimens from potential dissemination sites (e.g., BALF, pleural fluid, thrombus, or other body fluids) may yield valuable diagnostic clues. The pathogenesis and progression mechanisms of TWE warrant further investigation.

## Data Availability

The original contributions presented in the study are included in the article/Supplementary Material, further inquiries can be directed to the corresponding author.
