# Supplementary material for: Severe mitral valve papillary muscle rupture of isolated Whipple's endocarditis: a case report and review of the literature
Source: Front Cardiovasc Med. 2025 Nov 11;12:1669997. doi: 10.3389/fcvm.2025.1669997 (PMC12644038; doi:10.3389/fcvm.2025.1669997)
Supplement: Supplementary file 1 [file Datasheet1.pdf]

Supplementary materials: Literature search methods

Search Terms: "Whipple" or "Tropheryma" or "*Tropheryma whipplei* " and "endocarditis"

Inclusion Criteria: (1) Human studies; (2) Published in English; (3) Reporting data on patients' clinical characteristics, microbiology, treatment, and outcomes. (4) a confirmed diagnosis of Whipple's endocarditis by serological, valvular, or extracardiac tissue examination polymerase chain reaction (PCR), periodic acid–Schiff (PAS), or immunohistochemistry (IHC).

Exclusion Criteria: (1) Secondary research papers (e.g. reviews), editorials ; (2) Cases of classic Whipple's disease (WD), defined as those presenting with the clinical manifestations of classic WD (e.g., gastrointestinal symptoms, arthralgia, or unintentional weight loss) and, if available, a positive intestinal biopsy for *T. whipplei*; (3) papers not reporting results on primary research or insufficient data; (4) studies not in humans; (5) studies not in English; (6) No full text available

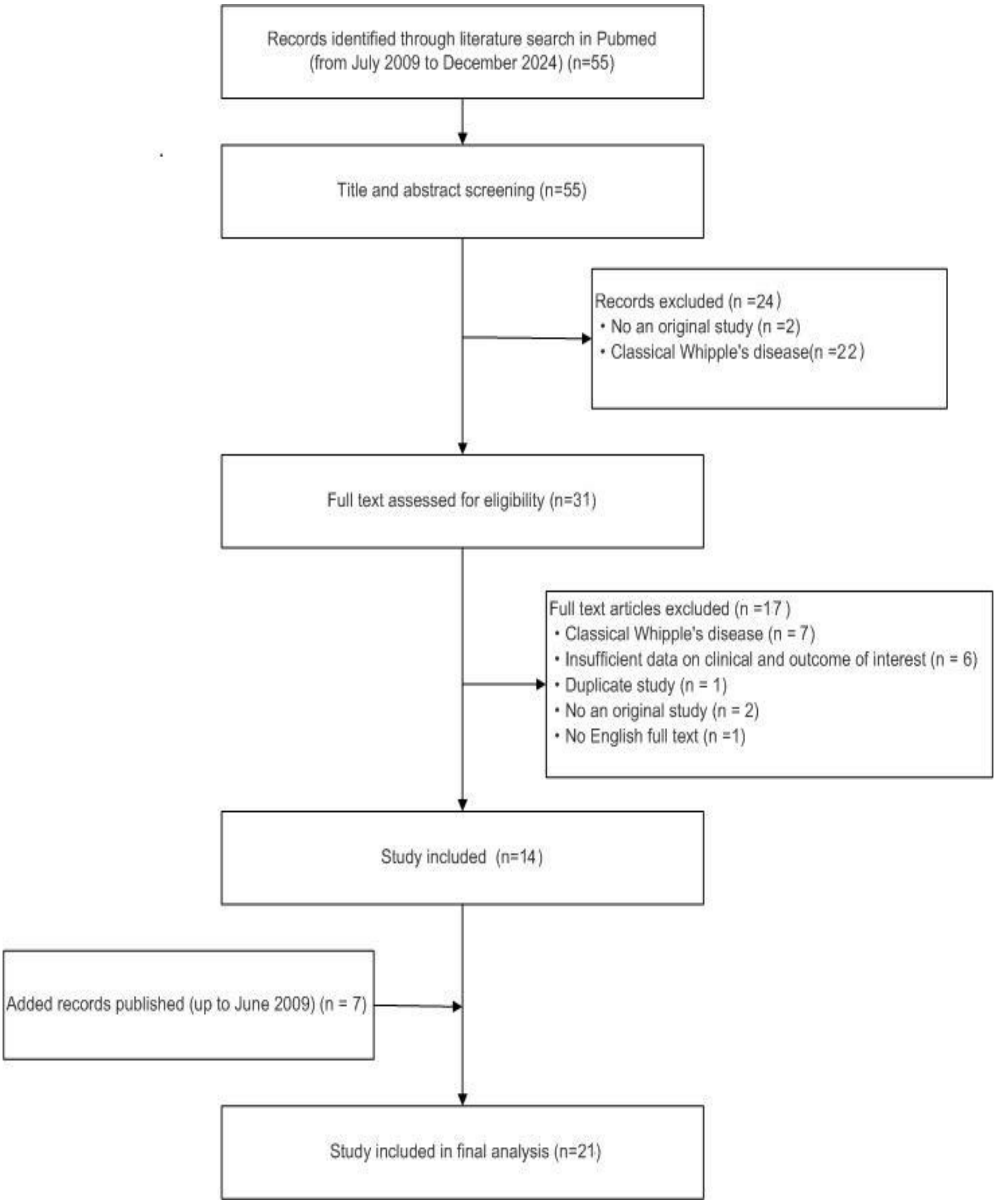

Figure S1. Flow chart of case selection in this study

Table S1. The clinical characteristics, diagnosis, and prognosis of 37 patients with isolated *T. whipplei* endocarditis

Case [Ref]	Age	Sex	IS	Presenting Symptom	Fever	Cardiac history	Alcohol abuse	Other Comorbidities	Involved valves	Vegetations	Valve analysis	Intestinal biopsy	Other methods	Treatment	Outcome
1[11]	78	M	N	HF	N	CHD	N	CRI	AV	Y	PCR+	ND	ND	Cef, 2 w; SXT 1 y	Well after 4.5 y
2[11]	70	M	N	HF	N	N	Y	COPD,PVD	MV	Y	PCR+	ND	ND	Cef, 2 w; SXT 1 y	Well after 2 y
3[11]	76	M	Y[a]	Stroke, MCA occlusion	N	CHF	N	HTN,CRI,Stroke	MV	Y	PCR+	ND	ND	Cef, 2 w; SXT 1 y	Well after 2 y
4[12]	58	M	N	HF	N	CMP	Y	No	AV+ MV	Y	PAS+ PCR+	PAS neg	ND	Intensive care support	Death POD 2
5[13]	78	M	N	HF	N	AP, CHF	N	No	MV	N	PAS+ PCR+	PAS neg	BM PAS neg	Cef and VAN	Well
6[14]	51	M	N	Multiple arteries emboli	N	Bicuspid AV	N	Gout, HTN	AV	Y	PCR+	ND	ND	VAN, 4w; SXT,12m	Well
7[15]	68	M	N	Valve dysfunction	-	CHD	N	N	AV	N	PCR+	PAS neg PCR neg	ND	Cef,3w; SXT,1y	Well
8[16]	51	M	N	HF	-	N	N	N	AV+MV	Y	PAS+ PCR+	PAS neg	ND	G,VAN and SXT; SXT,1y	Well
9[17]	62	M	N	Stroke	Y	N	N	HTN	AV+MV	N	PAS+	ND	ND	Mer 2 w; C 1 y	Well
10[18]	71	M	N	Non-STEMI	-	N	N	N	AV+MV	N	PCR+	ND	ND	SXT,1y	Well
11[19]	50	M	N	Presumed septic shock	N	N	Y	Cachexia	TV	Y	No surgery	ND	PCR of blood, pleural fluid +	Cef, 25d; D, H and SXT,1y	Well
12[20]	48	M	N	Stroke	N	N	N	N	AV	Y	PCR+ PAS+	ND	ND	Cef,6w; SXT,14m	Well
13[21]	80	M	N	Stroke	N	N	N	N	AV	Y	PCR+	ND	ND	Cef, 6 w; D and H, 1y	Well

14[22]	67	M	N	HF	N	MVP	N	N	MV	Y	PAS+ PCR+	ND	ND	Cef, 2w; SXT,1y	Well
15[23]	57	M	N	Femoral arteries Emboli	N	N	N	PVD	AV	Y	PAS+ PCR+	ND	ND	-	Death POD 2
16[23]	48	M	N	STEMI	N	-	N	N	AV	Y	PCR	PAS neg	ND	LVAD; Cef+VAN, 4w; SXT, 1y	Heart transplant after 2y
17[24]	77	F	N	Stroke	N	PV	N	N	AV	Y	PCR	ND	ND	D and H,18 m	Well
18[25]	70	F	N	Stroke,MCA occlusion	Y	N	N	N	AV	±	No surgery	ND	Thrombus aspirate PCR+	NA	Vegetation disappeared at the 3 m follow-up
19[26]	51	M	Y[a]	HF	Y	N	N	COPD;CRI	AV	Y	PCR	ND	ND	Empiric VAN and G	Death POD 5 due to vasoplegia
20[27]	63	F	N	limb arterial emboli	N	N	N	N	AV	Y	PCR	ND	ND	Cef,6w; D and H,1y	Well
21[27]	62	M	N	ACS	N	N	N	N	AV	Y	PCR +	ND	ND	Cef 6w; D and H,1y	Well
22[28]	80	F	N	PV failure	N	PV	N	N	AV	N	PCR+	ND	ND	Cef 2w; SXT,1 y	Well
23[29]	50-60	M	Y[a]	limb arterial emboli	N	N	Y	N	AV	Y	PCR+	NA	ND	Empiric VAN, D and P/T	Death shortly after surgery
24[5]	62	M	N	HF	N	AVI; PIE; PV	N	CVA	AV+MV	Y	PAS+ PCR+	ND	ND	Cef, 2w; SXT,1y	Well
25[5]	56	M	N	HF	N	N	Y	HTN, CLD	AV	Y	PCR+	ND	ND	Cef,2w; SXT,1y	Well
26[5]	71	M	N	HF	N	N	Y	N	AV	Y	PCR+	ND	ND	Cef,2w; SXT,1y	Well
27[5]	79	M	N	HF	N	AVI	N	HTN, CRI	AV	N	PCR+	ND	ND	G; SXT,1y	Well
28[5]	62	M	N	HF	N	N	Y	N	AV	N	PCR+	ND	ND	SXT,1y	Well
29[5]	49	M	N	HF	N	N	N	N	AV+MV	Y	PCR+	ND	ND	Cef, 2w; SXT,1y	Well

30[5]	54	M	N	HF	N	N	N	N	MV	Y	PCR+	ND	ND	H+SXT(ongoing)	Well
31[5]	50	M	N	HF	Y	AVI	N	CVA	AV+MV	N	PCR+	ND	ND	SXT, 40m	Well
32[6]	45	M	N	Stroke	Y	BAV	N	N	AV	Y	PCR+	PAS neg	ND	AMX+GEN,15d; D+H(ongoing)	Well
33[6]	56	M	N	HF	Y	CS	N	N	AV	Y	PCR+	PAS neg	ND	C+G15d; D+H(ongoing)	Well
34[6]	51	M	N	Stroke	N	N	N	N	AV	Y	PCR+	PAS neg	ND	AMC+GEN,11d; D and H(ongoing)	Well
35[6]	50	M	N	HF	N	N	N	N	MV	N	PCR+	PAS neg	ND	Cef, 5d; D and H(ongoing)	Well
36[6]	71	M	N	HF	N	N	N	N	AV	Y	PCR+	PAS neg	ND	Cef+G,15d; D+H,1.5y	Well
37[6]	68	M	N	HF	Y	N	N	N	AV	Y	PCR+	PAS neg	ND	Cef+G,15d; D+H,1y	Well

[a] The underlying diseases of immunosuppression in nos. 3, 19, and 23 were prostate cancer, radiotherapy and chemotherapy for Hodgkin's lymphoma, and intravenous drug use, respectively.

Abbreviation: ACS, acute coronary syndrome; AM, amoxicillin–clavulanate; AMX, amoxicillin; AP, Angina pectoris; AV, Aortic valve; AVI, Aortic valve insufficiency; BAV, bicuspid aortic valve; BM,bone marrow; BRA, Branch retinal artery; Cef, ceftriaxone; CMP,Cardiomyopathy; CHC, Chronic hepatitis; CHD, Coronary heart disease; CHF,congestive heart failure; CLD,chronic lung disease; CRI,chronic renal insufficiency; CS, coronary stent; CVA,cerebrovascular accident; D,Doxycycline ; G, gentamicin; H, hydroxychloroquine; HF, heart failure; HTN, hypertension; HL, Hodgkin's lymphoma; IS, immunosuppression; LVAD, Left Ventricular Assist Device; m, month(s); MCA, Middle cerebral artery; Mer, meropen; MR, mitral regurgitation; MV, mitral valve; MVP, mitral valve prolapse; neg, negative; ND, Not done; PAS, periodic acid-Schiff-diastase staining; PG, Penicillin G; PIE, previous infective endocarditis; PMR, polymyalgia rheumatica ; POD, postoperative day; P/T, piperacillin/tazobactam; PV, prosthetic valve; PVD, peripheral vascular disease; RA, rheumatoid arthritis; SXT, trimethoprim and sulfamethoxazole; STEMI, ST-elevation myocardial infarction; TV, tricuspid valve; Van, vancomycin; w, week(s); y, year(s); +: positive; –, not reported; ±, suspicious.
